# Overlooked potential of positrons in cancer therapy

**DOI:** 10.1038/s41598-021-81910-4

**Published:** 2021-01-28

**Authors:** Takanori Hioki, Yaser H. Gholami, Kelly J. McKelvey, Alireza Aslani, Harry Marquis, Enid M. Eslick, Kathy P. Willowson, Viive M. Howell, Dale L. Bailey

**Affiliations:** 1 School of Physics, Faculty of Science, The University of Sydney, Sydney, Australia; 2 Department of Nuclear Medicine, Royal North Shore Hospital, Sydney, Australia; 3 Bill Walsh Translational Cancer Research Laboratory, Faculty of Medicine and Health, The University of Sydney, Sydney, Australia; 4 Sydney Vital Translational Cancer Research Centre, Sydney, Australia; 5 Faculty of Medicine and Health, The University of Sydney, Sydney, Australia

**Keywords:** Radiotherapy, Targeted therapies, Biological physics

## Abstract

Positron (β^+^) emitting radionuclides have been used for positron emission tomography (PET) imaging in diagnostic medicine since its development in the 1950s. Development of a fluorinated glucose analog, fluorodeoxyglucose, labelled with a β^+^ emitter fluorine-18 (^18^F-FDG), made it possible to image cellular targets with high glycolytic metabolism. These targets include cancer cells based on increased aerobic metabolism due to the Warburg effect, and thus, ^18^F-FDG is a staple in nuclear medicine clinics globally. However, due to its attention in the diagnostic setting, the therapeutic potential of β^+^ emitters have been overlooked in cancer medicine. Here we show the first in vitro evidence of β^+^ emitter cytotoxicity on prostate cancer cell line LNCaP C4-2B when treated with 20 Gy of ^18^F. Monte Carlo simulation revealed thermalized positrons (sub-keV) traversing DNA can be lethal due to highly localized energy deposition during the thermalization and annihilation processes. The computed single and double strand breakages were ~ 55% and 117% respectively, when compared to electrons at 400 eV. Our in vitro and in silico data imply an unexplored therapeutic potential for β^+^ emitters. These results may also have implications for emerging cancer theranostic strategies, where β^+^ emitting radionuclides could be utilized as a therapeutic as well as a diagnostic agent once the challenges in radiation safety and protection after patient administration of a radioactive compound are overcome.

## Main

**Figure 1:**
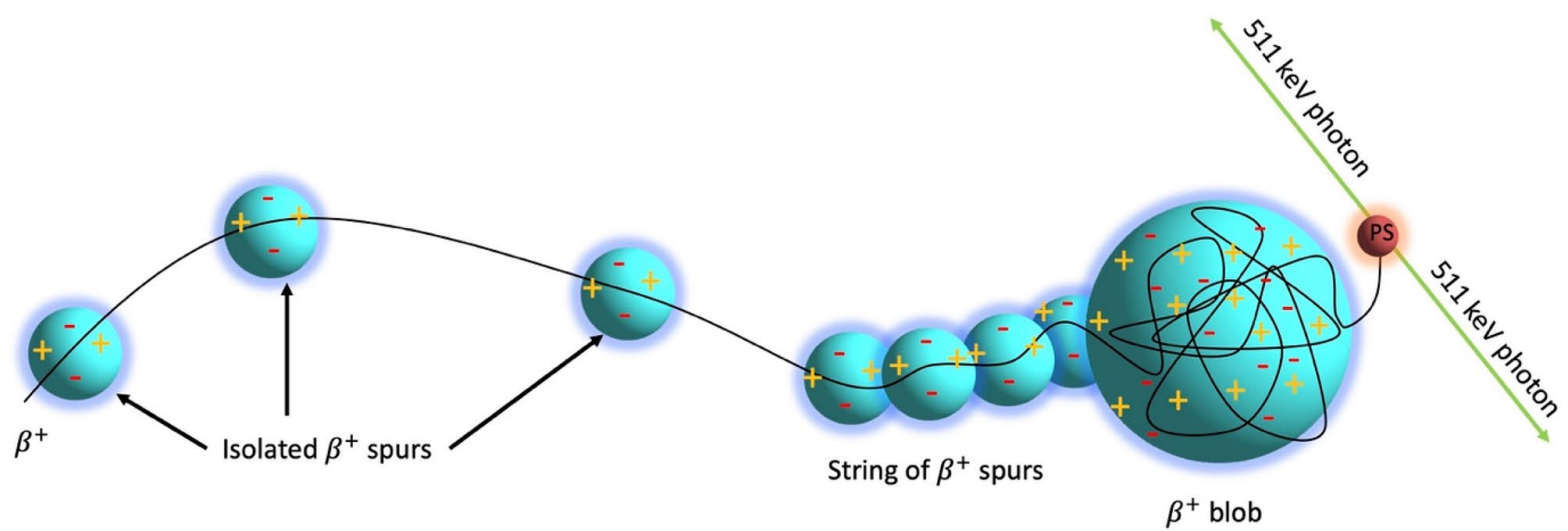
β^+^ track deposits energy in water in through the formation of “spurs” and the terminal “blob”, before a positronium (Ps) formation and annihilation to generate the 0.511 MeV photons.

Approximately 90% of cancer related deaths are from metastatic disease and thus the focus of cancer radiotherapy (RT) in both the scientific and clinical communities is shifting towards a more targeted, personalized approach^1^. While the radiobiology of gamma, MV X-rays and particles (e.g., β^−^ α^2+^, Auger electron) for therapy have been well investigated, there is a paucity of in vitro and in vivo studies on the biological impact of the positron (β^+^) which could provide a new modality for cancer RT^2–5^. The β^+^ is the anti-matter counterpart of the electron and its emission from a radionuclide such as fluorine-18 (^18^F), leads to the conversion of a proton within the atomic nucleus to a neutron accompanied by a release of an electron neutrino. This decay process results in two 0.511 MeV photons due to the annihilation of the β^+^ when coming to rest in matter and making contact with an electron. β^+^ lose their kinetic energy in discrete quantities (≈ 100 eV) producing ‘rabbles’ of excited and ionized molecules^6^. They create an excess-electron/positive-ion pairs inside a spherical nano-volume along its radiation track (called the β^+^ ‘spur’) and during the post-thermalization process (called the terminal β^+^ ‘blob’) with further annihilation ionization when they come to rest after positronium (Ps) formation (Fig. 1)^7–9^.

Each spur and blob on the particle track are a temporal and spatial microcosm of highly reactive species capable of locally depositing energies up to 100 and 5000 eV respectively^9^. Thus, a relatively large radiation dose (i.e., large energy deposition in a small volume) can be delivered when a spur or blob interacts with biomolecules such as DNA^9^. Despite these unique radiation properties, the fundamental interactions of β^+^ particles before the annihilation process and its potential therapeutic impact in vivo/vitro, have not been considered due to its prominent role in PET to image annihilation photons. The aim of our study was to derive the radiobiological parameters for ^18^F β^+^ emission and demonstrate proof-of-concept cytotoxicity data for β^+^ emission in radionuclide therapy (RNT). These results may have implications for novel RNT treatment planning strategies, where β^+^ emitting radionuclides are used as a theranostic.

**Figure 2:**
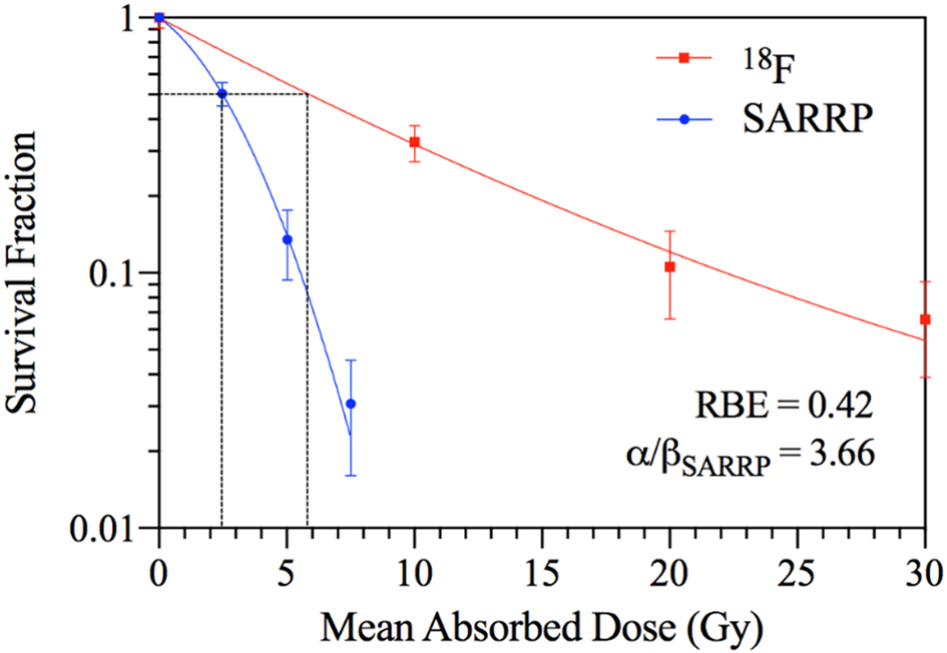
LNCaP C4-2B Clonogenic Cell Survival: ^18^F β^+^ emission versus SARRP EBRT. Black dotted line depicts the mean absorbed dose required to reduce cell survival fraction to 0.5.

Using clonogenic assays described in the methods, the cell survival fraction (SF) shown in Fig. 2 demonstrated > 90% cell kill when LNCaP C4-2B prostate cancer cells were treated with 20 Gy ^18^F β^+^. Furthermore, Fig. 2 shows the relationship between the absorbed dose of ^18^F β^+^ emission and its impact on cell clonogenicity compared to X-ray external beam radiotherapy (EBRT) using the small animal radiation research platform (SARRP—See supplementary data). Both data have a linear quadratic (LQ) trend fitted for the purpose of visualization and to extrapolate the relative biological effectiveness (RBE). The SF data from ^18^F are relatively linear up to 20 Gy, but trends towards an exponential decay at higher doses. SARRP X-ray irradiation induced an expected LQ trend in cell kill, with a shoulder at ~ 2.5 Gy, and a linear region at higher doses [α/β ratio = 3.66; Eq. (3)]. The calculated mean RBE from Fig. 2 was 0.42 for ^18^F relative to the 0.5 SF for the SARRP.

**Figure 3:**
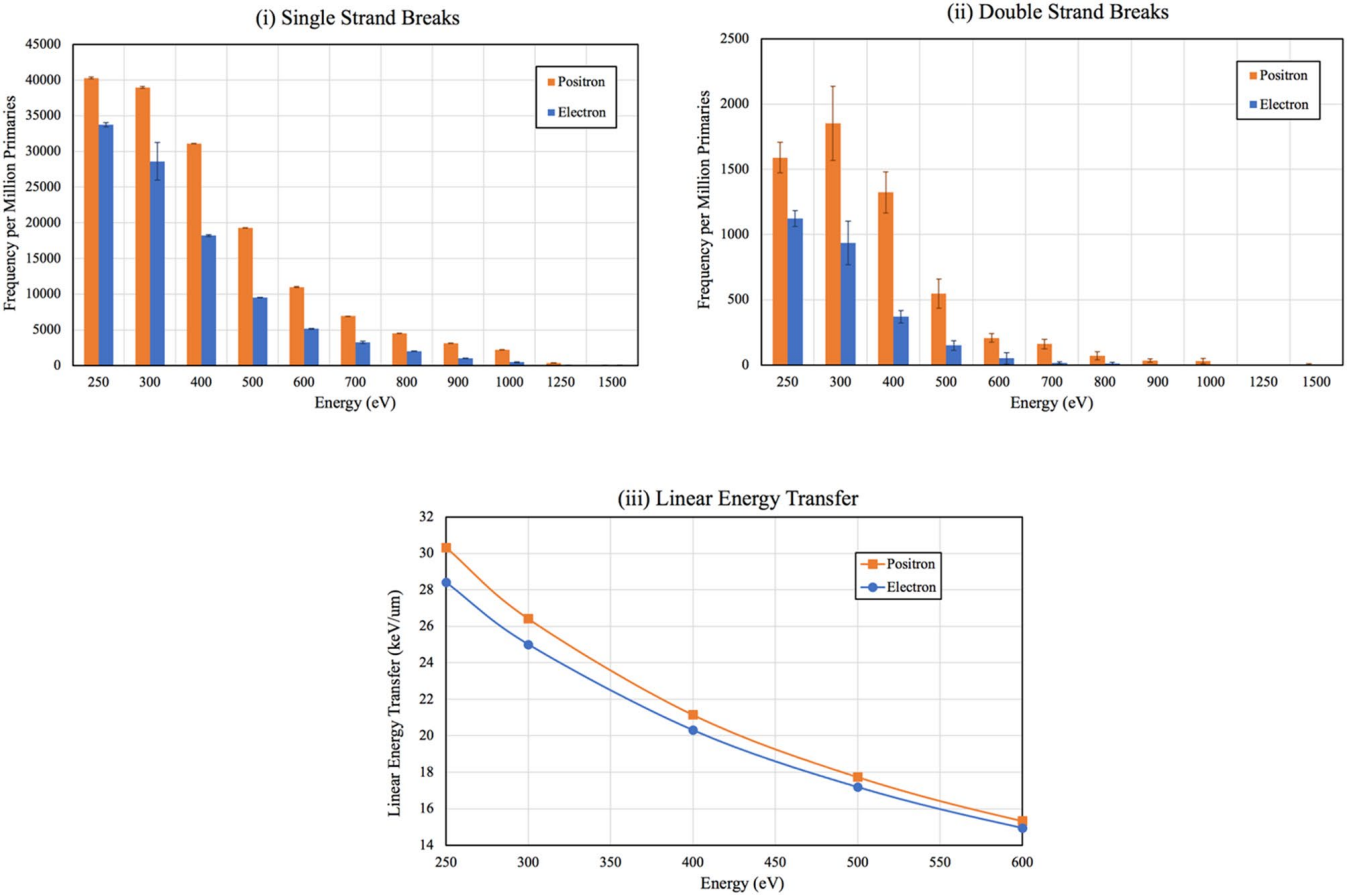
(i) SSB frequency and (ii) DSB frequency for β^+^ and electrons from the TOPAS-nBio simulation. The highest difference in the frequency between β^+^ and electrons occurred at 400 eV. (iii) Plot of the primary β^+^ and electron LET (keV/μm) across the simulated energies.

Using Monte Carlo (MC) simulation of a linear DNA model in TOPAS-nBio we acquired the frequency of hits per one million primaries resulting in single strand breaks (SSBs) and double strand breaks (DSBs) from the emission of β^+^ and β^−^/electrons, presented as a function of the particle’s kinetic energy in Fig. 3(i),(ii)^10,11^. Both β^+^ and β^−^/electrons exhibited a similar trend for SSB with an exponential decrease for particles with higher kinetic energies (Fig. 3(i)). DSB frequency for both β^+^ and electrons showed an exponential trend as their energy increased (Fig. 3(ii)). The high fluctuations in the recorded DSB for energies < 400 eV is primarily due to the limitations of the MC package PENELOPE, as the particle transport is not recorded below 100 eV. The spatial dependency of a DSB also contributes to the fluctuations, as variations in SSBs for a particular energy are significantly lower than that of DSBs. Observing the trend of the plot for the linear energy transfer (LET) (Fig. 3(iii)) for the primary particle simulated, the expected maximum SSB and DSB frequency for both β^+^ and electron would have been at 250 eV for the simulated energies. Although the LET of β^+^ and electrons significantly increase when they come to rest, β^+^ have relatively higher LET (≈7% higher at E = 250 eV).

This is also consistent with the blob model where the β^+^ creates an excess-electron/positive-ion pairs towards the end of its track. In addition to the β^+^ annihilation ionization with outermost valence electrons (which is the predominant process), a fraction of sub-eV β^+^ can tunnel through the repulsive nuclear potential and annihilate with core electrons (a phenomena used in Induced Auger Electron Spectroscopy)^12^. This creates a vacancy in the inner electronic shell leading to an emission of high LET Auger electrons and, thus, a β^+^ traversing the DNA has a higher probability of causing lethal damage when compared to electrons. This is again consistent with the trend observed for both the simulated SSB and DSB induced by β^+^ tracks.

The greatest difference in SSBs and DBSs for the emission profiles occurred at 400 eV where β^+^ demonstrated a 55% integral increase in SSBs compared to electrons, and a 117% increase in DSBs. These results are consistent with our previous study demonstrating the use of ^89^Zr and ^64^Cu for dose enhancement in nuclear medicine imaging compared to β^−^ therapeutic isotopes such as ^67^Cu, by virtue of their higher ionization properties^13^. Another in vivo study demonstrated that both ^64^Cu (with 38.5% β^−^) and ^67^Cu (with 100% β^−^) were an effective targeted β^−^ RNT for subcutaneous human colon carcinoma carried in hamster thighs when radiolabeled with a mouse anti-human colorectal cancer^14^. Although in this study only the β^−^ emission of ^64^Cu (with decay branching ratio: 38.5% β^−^, 43.9% electron capture and 17.6% β^+^ emission) was considered for therapy, our results suggest that there could be a noticeable contribution of β^+^ emission in the therapeutic effectiveness of ^64^Cu targeted RNT.

Cell survival curves of X-ray versus β^+^ irradiation of LNCaP C4-2B cells revealed substantial cell kill (≈ 70% at 10 Gy) by ^18^F β^+^ emission. This may be therapeutically significant as previous in vitro studies have shown less than ~ 30% cell kill in a range of cancer cell lines by 10 Gy of β^−^ emitting radionuclides, ^90^Y and ^177^Lu^15,16^. This suggests that the RBE of ^18^F β^+^ emission may be three times higher than β^−^ emitters depending on the β^−^ and β^+^ emitters being compared. Although these results suggest that other β^+^ emitter isotopes such as ^68^ Ga, ^64^Cu and ^89^Zr could potentially be considered as a therapeutic isotope, due to their differences in positron energy spectrum, branching ratio and half-life, further in vitro experiments are required to quantify their therapeutic impact. The MC simulation demonstrated 1.5-fold and 2.2-fold more SSBs and DSBs, respectively, are induced by β^+^ tracks, when compared to electron tracks. Thus, the direct interaction of a single β^+^ with DNA results in more lethal (i.e., DSB) damage than for a single electron.

Based on the spur model (Fig. 1), a β^−^/electron and β^+^ have similar radiation tracks except at sub-keV energies^7–9^. For a sub-keV electron, the mean separation between two energy deposition events and the formation of spurs (≈ 40 nm) is 20 times larger than the diameter of the DNA helix (≈ 2 nm)^7,16^. However, for a sub-keV β^+^ track (i.e., thermalized β^+^), spurs are formed in a continuous distribution of radiation damage with a higher density of several electrons and ions. Thus, at sub-keV energies, β^+^ have a higher LET compared to β^−^/electrons (Fig. 3(iii))^6,7^. In addition, the total number of ionizations per β^+^ track is greater than per electron due to the additional ionization occurring at the terminal annihilation event. Moreover, in a β^+^ spur the incident β^+^, secondary electrons and ions are within each other’s electrostatic/Coulomb field resulting in greater localization of energy deposition and a higher probability of inducing direct DNA DSB.

As per convention, the radiobiological parameters of ^18^F β^+^ emission in our in vitro study were benchmarked against X-ray therapy. The low mean β^+^ RBE of 0.42 compared to X-ray (SARRP) calculated from the cell survival curve can be attributed to the > 200-fold difference in dose rate between the two modalities (i.e. ≈ 7–21 mGy/min and 4.5 × 10^3^ mGy/min respectively). For X-rays, the main mechanism of cell kill is through indirect methods primarily via the production of free radical species.

X-ray EBRT cell survival fraction curves often have a shoulder [quadratic region of LQ model; Eq. (3)] at 2 Gy contributed by the reconciliation of DNA damage and DNA repair^1,2^. In LNCaP C4-2B cells this was observed at 2.5 Gy (Fig. 2) using the SARRP^1,2^. While the lower initial slope observed for ^18^F β^+^ irradiation at this similar dose region may be due to dose rate differences between SARRP and ^18^F irradiation, at the higher doses, the clonogenic survival approaches a deterministic process where the relative difference in number of surviving clonogenic cells is proportional to the increase in dose [linear region of LQ; Eq. (3)], and thus the corresponding survival curve becomes exponential^17^.

Current administration of β^+^ emitting radionuclides in the clinic ensures that the doses absorbed to the patients in diagnostic nuclear medicine are minimized and reported values for mean effective doses for ^18^F-FDG range from 3.4 to 13.4 mSv^18^. In RNT, therapeutic doses vary significantly due to differences in tumor type, burden and administration method. For example, absorbed doses range from 1.2 to 540 Gy in thyroid cancer patients, whereas for prostate cancer reported doses range from 3.4 to 92.5 Gy^19,20^. The large differences between diagnostic and therapeutic doses in part explains the lack of investigation into β^+^ emitters as a therapeutic agent. The abundance of currently available targeting ligands for imaging using β^+^ emitters allow for a “theranostic” application with the same radionuclide to be feasible using this approach^21^.

Our study presents the first in vitro evidence of therapeutic effect of β^+^ emitters in cancer medicine, with an attractive prospective for future studies. Clinically, the highly penetrating, low ionizing properties of the emitted 0.511 MeV photons mean that the safety and logistics behind the administration of therapeutic doses of β^+^ emitters must be reconsidered. Although a specialized form of automated/remote injection in a shielded room will be required, the relatively short half-life, cost, availability and reduced imaging time is justifiable to find the means of overcoming the limitations presented.

## Methods

### Cell culture

The human prostate cancer cell line LNCaP C4-2B, (ATCC, Catalogue no. CRL-3315), was cultured in Roswell Park Memorial Institute (RPMI) medium supplemented with 10% v/v fetal bovine serum (FBS) and incubated in a humidified atmosphere containing 5% CO_2_ at 37 °C.

### SARRP X-ray irradiation

LNCaP C4-2B cells were grown in T-25 flasks in 5 mL of medium for 3 days prior to the irradiation to ensure approximately 80% confluence of the cells in the flask. Using the SARRP (Xstrahl Inc, Atlanta, Georgia, USA), the EBRT open-field setup (20 × 20 cm^2^) was utilized. Flasks were placed at the isocenter of the beam on top of a 3.6 cm Perspex backscatter base at a source-to-axis distance (SAD) of 33.4 cm to give a dose rate of 4.45 ± 0.06 Gy/min at the flask with the default X-ray beam energy spectrum of 225 kVp. Doses using this setup were confirmed using EBT3 GAFchromic™ films. Doses ranging from 0 to 7.5 Gy in increments of 2.5 Gy were delivered by changing the exposure time according to the dose rate. The cells were then harvested for seeding post-irradiation.

### ^18^F irradiation

^18^F has a half-life of 109.77 min with a branching ratio of 96.73% β^+^ emission and 3.27% electron capture^22^. The emitted β^+^ has a mean energy of 249.8 keV and a maximum energy of 633.5 keV, corresponding to mean ranges of approximately 0.6 and 2.4 mm in water, respectively^22,23^. The sodium fluoride (^18^F) solution was obtained from Cyclotek NSW (batch number: F181362001) with an activity concentration of 4.14 GBq/mL.

LNCaP C4-2B cells were grown in an identical manner for the SARRP and ^18^F irradiations. Doses were calculated analytically using Eqs. (1) and (2) and were verified in silico using the sphere model (2 cm^3^ volume) in the OLINDA/EXM software Version 1.1 (Table 1)^24^.

1 $$D = \frac{{\tilde{A} \times \overline{E } \times \kappa }}{m}$$

2 $$\tilde{A} = A_{0} \mathop \smallint \limits_{0}^{T} e^{ - \lambda t} dt$$

where *D* (Gy) is the dose delivered, $\tilde{A}$ (Bq) is the cumulative activity, $\overline{E}$ (J) is the average energy of the emitted particle,$\kappa$ is the branching ratio (0.9673), *m* (kg) is the mass of the target volume (2 mL), $A_{0}$ (Bq) is the initial activity, *T* (s) is the irradiation time and *λ* (s^−1^) is the decay constant.

**Table 1:** Dose calculations for the administered ^18^F activities as determined from experimental (analytical) and in silico data (OLINDA/EXM).

Measured A_0_ (MBq)	Analytical positron dose (Gy)	OLINDA dose (Gy)
52.4 ± 0.5	9.7 ± 0.1	10.1 ± 0.1
104.9 ± 1.1	19.4 ± 0.2	20.1 ± 0.2
157.3 ± 1.6	29.1 ± 0.3	30.2 ± 0.3

The initial activity concentrations to achieve 10–30 Gy after 10 half-lives (≈ 18 h) ranged from 26.2 to 78.6 MBq/mL. Stock activity of ^18^F was diluted in an appropriate volume of medium to achieve the required initial activity for each analytical dose including a buffer time, confirmed using a dose calibrator with identical setup. Dose calibration confirmed the specific initial activity concentration of the radionuclide solution (at pH ≈ 7) and approximately 1% of the initial activity remaining after 18 h. For irradiation, the ^18^F medium solution was passed through a 0.22 μm filter to remove biological contaminants and then administered to the T-25 flasks once solutions reached the desired initial activity including buffer time. After 18 h, the radioactive medium was removed, and cells were washed with phosphate buffered saline (PBS) before harvesting for seeding post-irradiation.

### Clonogenic assay

Cell survival was determined using the clonogenic assay post-irradiation delayed plating (DP) method for both the SARRP and ^18^F experiments.^25^ The protocol used is further detailed in the “Supplementary Methods”.

### Radiobiological parameters

Using PRISM8 (GraphPad, v8.4.0, San Diego, California, USA), cell survival data from the SARRP irradiation were analyzed, and the LQ model [Eq. (3)] was fitted to calculate the radiobiological linear *α* (Gy^−1^) and quadratic components *β* (Gy^−2^) from the dose delivered D (Gy)^17^.

3 $$SF = e^{{ - \left( {\alpha D + \beta D^{2} } \right)}}$$

To calculate the RBE of ^18^F irradiation, the cell survival data from the radionuclide experiments were plotted against the EBRT SARRP data, and the RBE determined at the point of 0.5 cell survival fraction. The protocol used is further detailed in the “Supplementary Methods”.

### TOPAS-nBio simulation

**Figure 4:**
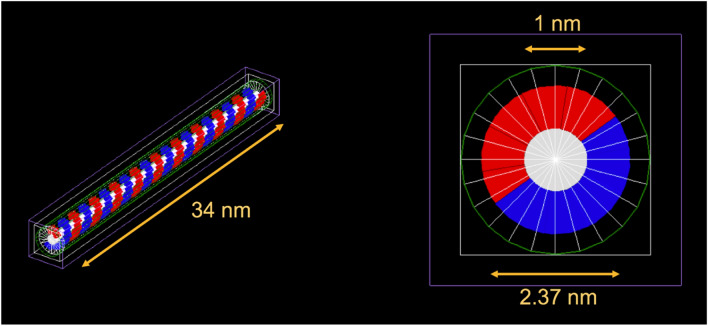
Linear DNA model in TOPAS-nBio, consisting of the sugar phosphate backbone (blue and red), bases (white) and volumetric source (green). 100 bases are simulated, with a total DNA length of 34 nm, outer and base diameter of 2.37 and 1 nm, respectively.

An MC simulation model was developed in the TOPAS-nBio toolkit to investigate the sub-cellular damage mechanism at DNA level by β^+^ and electron irradiations. All simulations were performed using TOPAS-nBio (version 1.0), which uses GEANT4 (Version 10.03.3)^10,11^. A standard linear DNA model (a half cylinder base and a quarter cylinder backbone) was simulated on this platform (Fig. 4) and isotropically irradiated with a volume source of β^+^ and electrons using energies ranging from 250 to 1500 eV to score the single and double strand breakages (SSBs and DSBs respectively)^11^.

Particles with energies above 1500 eV were not simulated as the cross-section for those energies is significantly small at DNA level. The number of primary incident particles (primaries) for the β^+^ and electrons was set to 10^6^, with 3 different seed numbers to gauge the spread in acquired values. The results are reported in frequency per million primaries for each energy. The strand breaks were scored by assuming an energy deposition threshold resulting in ionization of 17.5 eV, with a maximum of 10 bases between two SSBs to define a DSB^11^. The LET for the primary β^+^ and electron were simulated (GEANT4–NIST Database) in water for a range of sub-keV energies (250–600 eV).

## Supplementary Information

Supplementary Information.

## References

[1] Chaffer CL, Weinberg RA (2011). A Perspective on cancer cell metastasis. Science.

[2] Durante M, Orecchia R, Loeffler JS (2017). Charged-particle therapy in cancer: Clinical uses and future perspectives. Nat. Rev. Clin. Oncol..

[3] Nuhn P (2019). Update on systemic prostate cancer therapies: Management of metastatic castration-resistant prostate cancer in the era of precision oncology. Eur. Urol..

[4] Frey B (2014). Antitumor immune responses induced by ionizing irradiation and further immune stimulation. Cancer Immunol. Immunother..

[5] Pouget J-P (2011). Clinical radioimmunotherapy—The role of radiobiology. Nat. Rev. Clin. Oncol..

[6] Champion, C. & Loirec, C. L. Positron follow-up in liquid water: I. A new Monte Carlo track-structure code. *Phys. Med. Biol.* **51**, 1707–1723 (2006). 10.1088/0031-9155/51/7/005 16552099

[7] Mogensen OE (1995). Positron Annihilation in Chemistry.

[8] Stepanov SV (2012). Positronium in a liquid phase: Formation, Bubble state and chemical reactions. Adv. Phys. Chem..

[9] *Clinical radiation oncology*. (Elsevier, Amsterdam, 2016).

[10] Perl J, Shin J, Schümann J, Faddegon B, Paganetti H (2012). TOPAS: An innovative proton Monte Carlo platform for research and clinical applications: TOPAS: An innovative proton Monte Carlo platform. Med. Phys..

[11] Schuemann J (2018). TOPAS-nBio: An extension to the TOPAS Simulation toolkit for cellular and sub-cellular radiobiology. Radiat. Res..

[12] Chirayath VA (2017). Auger electron emission initiated by the creation of valence-band holes in graphene by positron annihilation. Nat. Commun..

[13] Gholami YH, Maschmeyer R, Kuncic Z (2019). Radio-enhancement effects by radiolabeled nanoparticles. Sci. Rep..

[14] Connett JM (1996). Radioimmunotherapy with a 64Cu-labeled monoclonal antibody: A comparison with 67Cu. Proc. Natl. Acad. Sci..

[15] Chan HS (2017). In Vitro comparison of 213Bi- and 177Lu-radiation for peptide receptor radionuclide therapy. PLoS ONE.

[16] Gholami, Y. H. *et al.* Comparison of radiobiological parameters for 90Y radionuclide therapy (RNT) and external beam radiotherapy (EBRT) in vitro. *EJNMMI Phys.* **5**, (2018). 10.1186/s40658-018-0217-8 PMC6119681 30175390

[17] Jones L, Hoban P, Metcalfe P (2001). The use of the linear quadratic model in radiotherapy: A review. Australas. Phys. Eng. Sci. Med..

[18] Quinn B, Dauer Z, Pandit-Taskar N, Schoder H, Dauer LT (2016). Radiation dosimetry of 18F-FDG PET/CT: Incorporating exam-specific parameters in dose estimates. BMC Med. Imaging.

[19] Sgouros, G. *et al.* Patient-Specific Dosimetry for 131I Thyroid Cancer Therapy Using 124I PET and 3-Dimensional–Internal Dosimetry (3D–ID) Software. 8. 15299063

[20] Violet J (2019). Dosimetry of ^177^ Lu-PSMA-617 in metastatic castration-resistant prostate cancer: Correlations between pretherapeutic imaging and whole-body tumor dosimetry with treatment outcomes. J. Nucl. Med..

[21] Chen K, Chen X (2011). Positron emission tomography imaging of cancer biology: Current status and future prospects. Semin. Oncol..

[22] Tilley DR, Weller HR, Cheves CM, Chasteler RM (1995). Energy levels of light nuclei A = 18–19. Nucl. Phys. A.

[23] Conti M, Eriksson L (2016). Physics of pure and non-pure positron emitters for PET: A review and a discussion. EJNMMI Phys..

[24] Stabin, M. G., Sparks, R. B. & Crowe, E. OLINDA/EXM: The Second-Generation Personal Computer Software for Internal Dose Assessment in Nuclear Medicine. 6. 15937315

[25] Franken NAP, Rodermond HM, Stap J, Haveman J, van Bree C (2006). Clonogenic assay of cells in vitro. Nat. Protoc..

