# Supplementary material for: Overlooked potential of positrons in cancer therapy
Source: Sci Rep. 2021 Jan 28;11:2475. doi: 10.1038/s41598-021-81910-4 (PMC7843622; doi:10.1038/s41598-021-81910-4)
Supplement: Supplementary file 1 — Supplementary Information. [file 41598_2021_81910_MOESM1_ESM.docx]

**Overlooked Potential of Positrons in Cancer Therapy**

Takanori Hioki^1,3,4^, Yaser H. Gholami^1,3,4^, Kelly J. McKelvey^3,4^, Alireza Aslani^2,5^, Harry Marquis^1,4^, Enid M. Eslick^2^, Kathy P. Willowson^1,2^, Viive M. Howell^3,4,5^ and Dale L. Bailey^2,4,5^

^1^School of Physics, Faculty of Science, The University of Sydney, Sydney, Australia

^2^Department of Nuclear Medicine, Royal North Shore Hospital, Sydney, Australia

^3^Bill Walsh Translational Cancer Research Laboratory, Faculty of Medicine & Health, The University of Sydney, Sydney, Australia

^4^Sydney Vital Translational Cancer Research Centre, Sydney, Australia

^5^Faculty of Medicine & Health, The University of Sydney, Sydney, Australia

**Supplementary Methods**

**Clonogenic Assay**

The DP method requires the harvesting and plating of cells after the initial DNA/cell repair processes are complete, typically > 6 hours post-irradiation.^25^ The time between irradiation and cell harvesting kept constant at 18 hours post-irradiation. Plated cells were then incubated for 7 days to allow for 6 to 7 cell cycles (LNCaP C4-2B doubling time is 24 hours) to form colonies of minimum 50 cells.^25^ After 7 days the medium was gently removed, cells washed with PBS and stained using 0.5 % w/v crystal violet in 50 % v/v methanol. After 15 minutes, the crystal violet stain was removed, cells washed with deionized water twice and dried overnight before imaging on a vSpot Spectrum EliSpot reader and analyzed using the colony counting software (Autoimmun Diagnostika GmbH, Straßberg, Germany). SF was calculated using equation (4).^25^

$SF= \frac{\text{No. Colonies: Irradiated Cells}}{\text{ No. Colonies: Control Cells}}$ (4)

**Radiobiological Parameters**

Doses delivered by SARRP were at a constant rate while ^18^F doses delivered with a constant Lea-Catcheside, G factor. Since the ^18^F dose rate mono-exponentially decays over the irradiation time, different ^18^F doses needed to be delivered with a constant G factor for RBE and SF comparison studies.^16^ A constant G factor assures an average constant DNA damage and repair rate for different doses of ^18^F. Therefore, different ^18^F doses with constant G factor can be comparable to doses delivered by X-rays at a constant dose rate, and the initial activities of ^18^F were dictated by keeping the G factor a constant. The G factor was calculated to be 0.35 using equation (5).^16^

$G= \frac{2}{\lambda-\mu} \left( \frac{\lambda}{1-e^{-\lambda T}} \right)^{2} \left( \frac{1-e^{-\left( \lambda+\mu\right)T}}{\lambda+\mu}- \frac{1-e^{-2\lambda T}}{2\lambda} \right)$ (5)

where the DNA repair time constant *μ* = $\frac{ln(2)}{\tau}$ and τ is the DNA repair half-life (30 minutes). Our previous paper discussed the relevance of using the extended LQ model for irradiation using radionuclides.^16^ The doses delivered were therefore calculated from the initial activity minus any residual activity. Due to the short half-life of ^18^F, the dose delivery was able to mimic a clinical administration of the radionuclide where the full decay of the radionuclide contributes to the total dose delivered.
